# In-vivo pharmacokinetic studies of Dolutegravir loaded spray dried Chitosan nanoparticles as milk admixture for paediatrics infected with HIV

**DOI:** 10.1038/s41598-022-18009-x

**Published:** 2022-08-16

**Authors:** Priya Dharshini K, Ramya Devi D, Banudevi S, Vedha Hari B. Narayanan

**Affiliations:** 1 Pharmaceutical Technology Laboratory, ASK-II, Lab No: 214, School of Chemical & Biotechnology, SASTRA Deemed-to-be-University, Thanjavur, Tamil Nadu 613401 India; 2 Centre for Nanotechnology and Biomaterials, School of Chemical & Biotechnology, SASTRA Deemed-to-be-University, Thanjavur, Tamil Nadu 613401 India

**Keywords:** Nanobiotechnology, Drug delivery, Drug development

## Abstract

Dolutegravir (DTG) is an antiretroviral drug approved in the year 2013, and being categorized as a BCS-II molecule, it possesses solubility issues. In order to enhance the solubility and improve its bioavailability, DTG-loaded Chitosan nanoparticles (NPs) were synthesized utilizing spray drying technology. The developed nanoformulation was characterized for its physicochemical properties and investigated for the feasibility of its administration through an oral route along with milk/food as an admixture for paediatric antiretroviral therapy. The in vivo oral bioavailability studies were conducted in Balb-C mice, where the animals were treated with the selected formulation of DTG-loaded Chitosan NPs and compared to pure DTG. The NPs exhibited 2.5-fold increase in the C_max_ (77.54 ± 7.93 μg/mL) when compared to the pure DTG (30.15 ± 8.06 μg/mL). This phenomenon was further reflected by the improved bioavailability of DTG (AUC: 678.3 ± 10.07 μg/h/mL) in the NPs administered to mice when compared to the AUC of animals administered with pure DTG (405.29 ± 7 μg/h/mL). Altogether, the research findings showed that Chitosan-based NPs were ideal carriers for oral administration of DTG along with milk and exhibited great potential to enhance the bioavailability of the drug and treatment adherence for paediatric HIV patients.

## Introduction

AIDS has been declared one of the most distressing health challenges in the world, which is caused by a specific viral agent human immune deficiency virus (HIV)^1^. The infection with HIV directly or indirectly attacks the human immune system, which causes the depletion of the CD4 T-cells and helper T-cells^2^. According to World Health Organization (WHO) statistics in 2020, it was estimated that about 37.7 million are infected with HIV, that includes 1.8 million children (age < 15 years)^3^. The antiretroviral therapy to treat HIV patients covers five classes of anti-HIV drugs (entry inhibitor, fusion inhibitor, reverse transcriptase inhibitor, integrase inhibitor, protease inhibitor)^4^. Dolutegravir (DTG) is an integrase strand transfer inhibitor that blocks the integration of the viral DNA into the host cell DNA, which is considered one of the prime steps in the lifecycle of HIV^5^ and thus inhibits the proliferation of the virus inside the host. DTG possesses several remarkable benefits like a once-daily-dose, a high genetic obstacle to drug resistance, and reduced drug-drug interactions. It is well tolerated and metabolically compatible when compared to other classes of ARVs^6^. Since it is a BCS class II drug, it exhibits limited aqueous solubility of 95 mg/L at 25 °C. The bioavailability of DTG in the cellular and tissue reservoirs is also reduced due to the presence of efflux transporters and drug-metabolizing enzymes, which leads to speedy elimination and restricted permeability^7^. Solubility enhancement of such drugs is an imperative part of drug delivery research due to the intrinsic struggle of developing it as a successful dosage form. To overcome the above-mentioned issue, various formulation techniques were established such as complexation, surface modification, nanoformulation, solid dispersion, etc.^8^.

Among the different methods, nanoformulation is considered one of the exceptional and expedient approaches, where the API is miniaturized to the nanometre range and hence the particles possess an increased surface area that leads to increased wettability, enhanced permeation, and uptake^9^. Due to their sub-cellular and sub-micron size, nanoparticles can penetrate deep into the tissues through fine capillaries, cross the physiological barriers, and are generally taken up efficiently by the reticuloendothelial cells^10^. The oral administration of the drug-loaded nanoparticles increases the cellular uptake, thereby enhancing the plasma exposure of the drug. NPs attain maximum availability by reducing the drug exposure to the enzymatic and non-enzymatic degradation in the GI tract and thereby avoiding first-pass metabolism and P-gp mediated efflux^11^. Therefore, nanoformulations could lead to an increase in AUC or drug exposure. Spray drying technology is been used for the past few decades to develop nanoparticles of various drugs^12^. Out of the various NPS, biodegradable polymeric NPs have been widely used in the pharmaceutical field. The advantages of using biopolymer include higher biocompatibility, reduced cost of medicine, lesser toxicity, higher therapeutic effect, improved bioavailability, etc.^13^.

Chitosan is a biocompatible, non-immunogenic, natural polycationic biopolymer, which is extensively used in the pharmaceutical industry. The presence of amino groups in the polymer backbone renders excellent properties such as controlled drug release, mucoadhesion, in situ gelation, transfection, permeation enhancement, efflux pump inhibitory properties, etc.^14^.

With this background, the current research deals with the synthesis and characterization of Dolutegravir-loaded Chitosan nanoparticles by spray drying technology. The comparative oral pharmacokinetics and organ biodistribution studies of DTG-loaded Chitosan NPs with free DTG were conducted in Balb-C mice to evaluate the in vivo performance of the developed nanoparticles.

## Results and discussion

### HPLC method validation

A rapid and sensitive HPLC method for the detection of DTG in biological samples was developed using a mixture of water, acetonitrile, and methanol in the ratio of 20:40:40 with 0.2% formic acid as the mobile phase. The developed method was validated for various parameters that are discussed as follows.

#### System suitability

**Figure 1:**
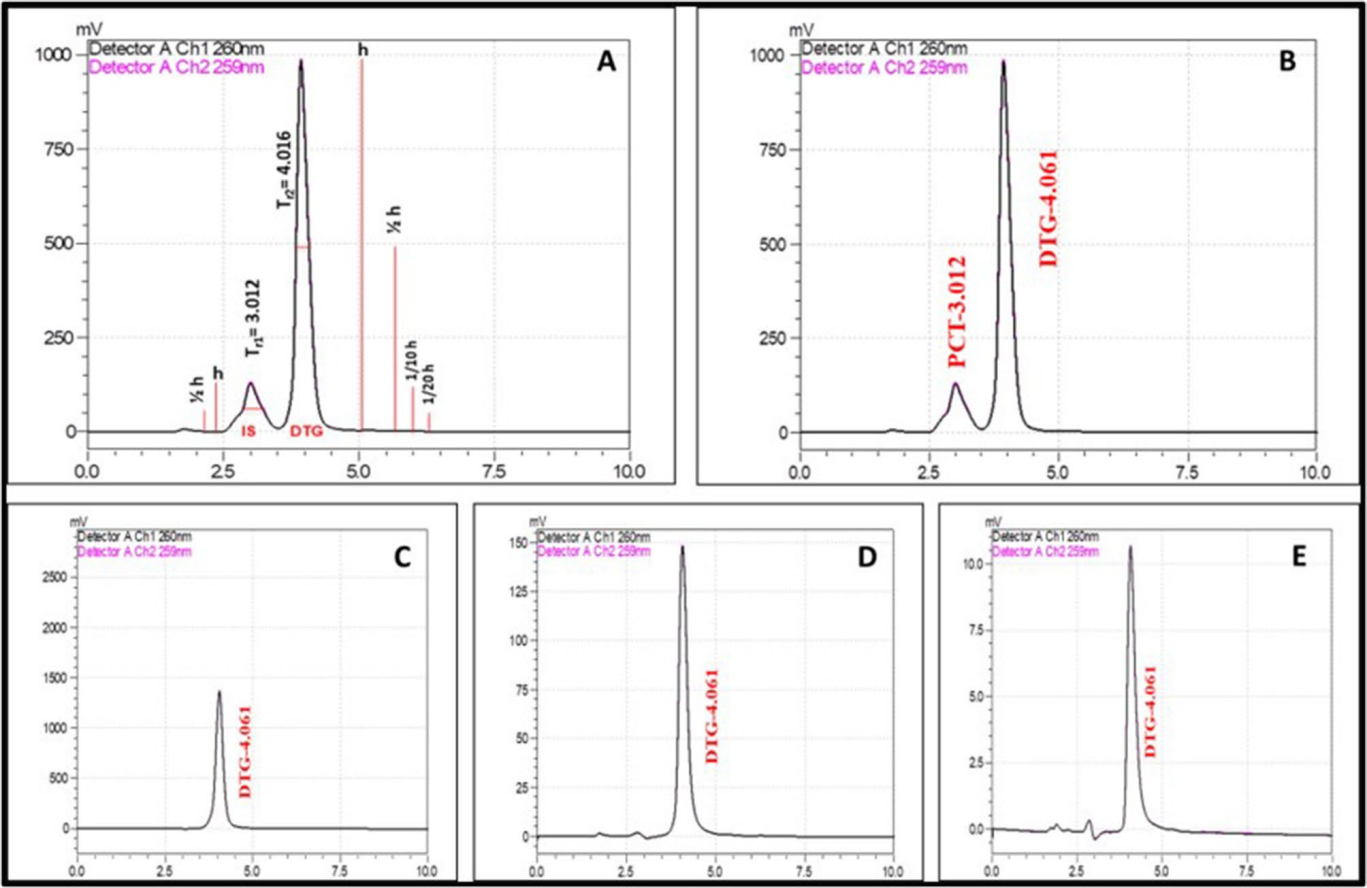
The comparative HPLC chromatograms of Dolutegravir developed using a mixture of water, acetonitrile and methanol in the ratio of 20:40:40 with 0.2% formic acid as the mobile phase. (**A**) System suitability; (**B**) Dolutegravir with internal standard; (**C**) low-level; (**D**) mid-level; (**E**) high-level of DTG concentrations.

System suitability is an important part of the method validation to evaluate the parameters like column efficiency calculation (N, 5.54(tr × w_0.5_)^2^), peak asymmetry factor (As, b/a @ 1/10 h), tailing factor (Tf, (a + b)/2a @ 1/20 h), theoretical plates (N × 100/Length of HPLC column), resolution (T_r2_ − T_r1_/[1.7 × 0.5(w_0.5_,1 + w_0.5_,2)]) and %RSD for replicate injections. The results were within the limits and were presented in Supplementary Table 1. Figure 1A showed the system suitability chromatogram (h—height of the peak; T_r1_, T_r2_—retention time of peaks 1 and 2).

#### Accuracy

The accuracy of the proposed method was found using the recovery studies. The known concentrations of pure drug were spiked in the placebo at three different levels i.e., 4 μg/mL (low, Fig. 1C), 40 μg/mL (mid, Fig. 1D), and 400 μg/mL (high, Fig. 1E). Accuracy was calculated as the percentage of the recovery. The results were tabulated in Supplementary Table 1.

#### Precision

The precision was assessed at three levels, reproducibility, repeatability and intermediate precision. Each level of precision was examined by six replicate injections at the concentration of 40 μg/mL Dolutegravir. The result of precision was expressed as % of RSD and was tabulated in Supplementary Table 1.

#### Linearity and range

The linearity was evaluated by measuring the different concentrations (100 ng–400 µg/mL) of the standard solutions of Dolutegravir. The calibration curve was constructed by plotting the concentration of standard solutions against mean peak areas and the regression equation was computed (Supplementary Fig. 1A–E,J).

#### Limit of detection (LOD) and limit of quantification (LOQ)

Estimation of LOD and LOQ is considered as the acceptable signal-to-noise ratios at 3:1 and 10:1, respectively. The limit of detection and quantitation of Dolutegravir was 8.25 and 24.91 ng/mL respectively.

#### Standard solution stability

The stability of the standard solution was tested for intervals of 24 and 48 h at room temperature. There were no significant changes observed in the system suitable parameters like theoretical plates, tailing factors, retention time, and resolution. Hence the standard solution is stable up to 48 h of room temperature.

#### Mobile phase stability

The stability of the mobile phase was tested for intervals of 24 and 48 h at room temperature. There were no significant changes observed in peak areas, theoretical plates, tailing factors, retention time, and resolution. Hence the mobile phase was proved to be stable up to 48 h of room temperature.

#### Calibration of the plasma spiked DTG with internal standard

The calibration curve Dolutegravir spiked in mice’s blood was shown in (Supplementary Fig. 1F–I,K). The curve exhibited linearity in the range of 1–10 µg/mL with a regression coefficient of 0.997. The internal standard (Paracetamol) was added to the spiked solution and the peak for the Internal Standard is obtained at 3.012 min, which confirmed the specificity of the proposed method (Fig. 1B).

### Physical characterization of Chitosan NPs

**Figure 2:**
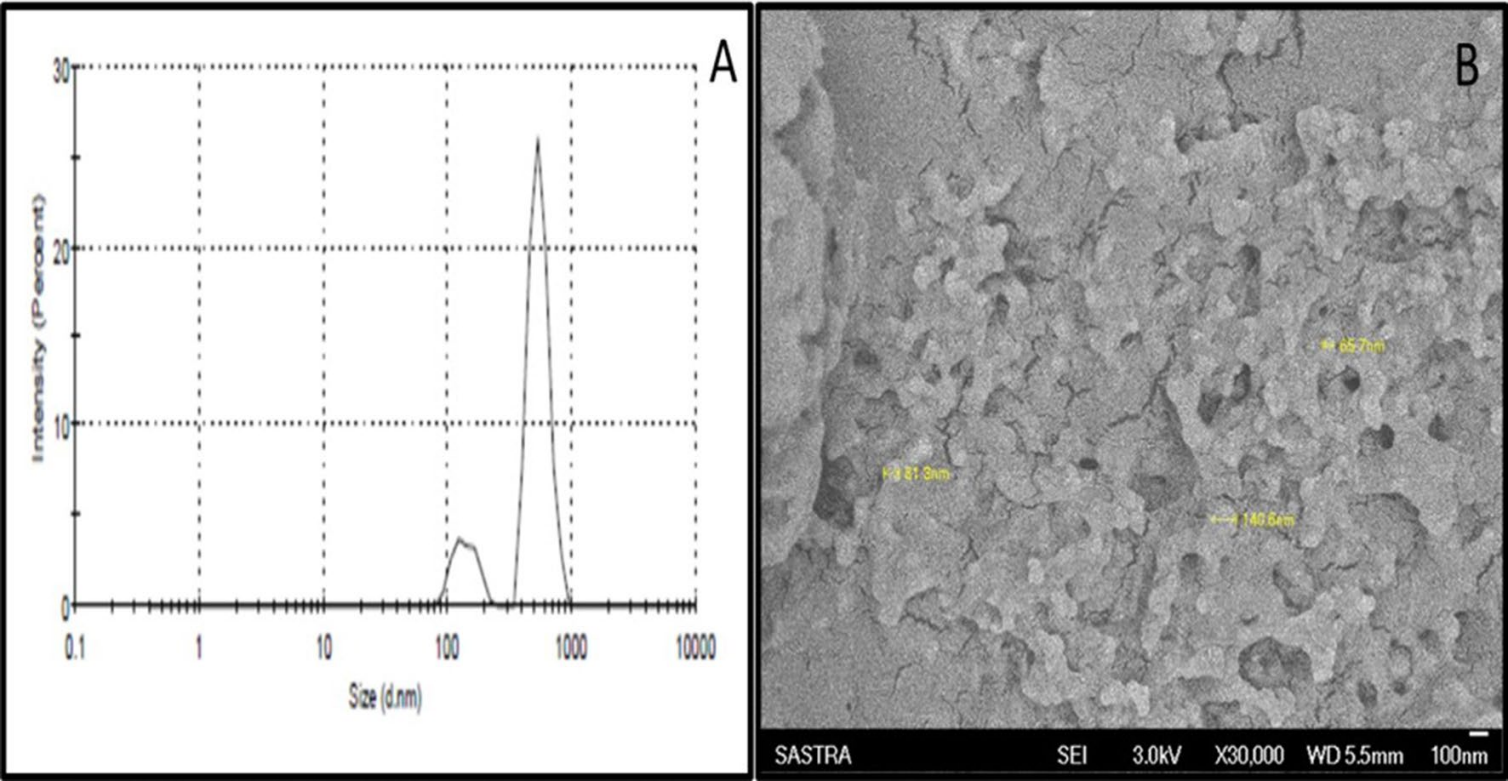
(**A**) Quasi elastic light scattering spectroscopy for the determination of particle size distribution. (**B**) Scanning electron microscopy for structure and morphology analysis (Magnification: × 30,000).

The DTG-loaded Chitosan NPs were subjected to particle size and SEM analysis to observe the morphological changes in the physical nature of the drug due to the spray drying process. The nanoparticles were spherical in shape post spray drying process (Fig. 2B). The size distribution of the nanoparticles was shown in Fig. 2A, wherein the majority of the particles were observed in the size range between 145 and 548 nm with a polydispersity index of 0.9. There were few particles that sized below 100 nm. Our results were similar to the PDI values ≥ 0.5 reported by Lazaridou et al., for the chitosan polymer-based spray-dried particles^15^. The results confirmed the feasibility of the spray drying process to produce nanosized particles without involving complex chemical processes.

### Chemical characterization of Chitosan NPS

The DTG-loaded Chitosan NPs were analyzed using FTIR and compared with the data of pure drug to confirm the chemical stability of the drug post spray drying process. The pure DTG exhibited peaks at 3434 cm^−1^ for the stretching bond of O–H, at 2976 cm^−1^ for C–H, and 1258 cm^−1^ for C–O–C bonds. The bending vibration peak of the C–H bond was observed at 856 cm^−1^. Further, the double bond stretching was noted at 1643 cm^−1^ and 1539 cm^−1^ for C=O and C=C, respectively. The characteristic C–N and C–F bonds in the pure DTG were displayed at 1023 cm^−1^ and 1060 cm^−1^ respectively^16^. All these characteristic peaks of DTG were observed in the drug-loaded Chitosan NPs with a very mild shift in the peak values. The peak shift was observed due to the mild interaction of the DTG with Chitosan during the drug entrapment in the Chitosan matrix. A similar phenomenon was reported by Li et al., wherein the Honokiol loaded Chitosan nanoparticles developed using the spray drying technique showed a mild peak shift in the FTIR spectrum^17^. The results of FTIR confirmed that the spray drying process and reaction with Chitosan did not alter the chemical nature of the drug (Supplementary Fig. 2).

To comprehend, the thermal behavior of DTG in Chitosan NPs after the spray drying process was analysed by DSC (Supplementary Fig. 2). The endothermic peak representing the melting peak (T_m_) was found at 359 °C for the pure DTG^18^. However, no endothermic peaks corresponding to the melting point of DTG were observed in drug-loaded Chitosan NPs. The absence of an endothermic peak in the nanoparticles could be ascribed to the solid-state conversion of pure DTG from crystalline form to amorphous form after the spray drying process in presence of chitosan. Our results are comparable to the previous reports by Chaudhary et al., wherein the endothermic peak corresponding to DTG was absent after encapsulating the drug inside the Poloxamer 407 polymer^7^.

### In-vitro drug release and kinetics of Chitosan NPs in 0.1 N HCl media

The drug content of the spray-dried Chitosan NPs was estimated with 5 mg of nanoparticles dissolved in 10 mL of 0.1 N HCl media. After incubation at room temperature for 1 h, the solution was centrifuged and the supernatant was subjected to UV–Visible spectrophotometry analysis to estimate the amount of drug present in the nanoparticles. The Chitosan nanoparticles showing the maximum drug content of 75 ± 2% were selected as the optimum formulation and results were previously published earlier^19^. Since, spray drying is a process that results in the formation of polymer-drug matrix nanoparticles formed by electrostatic interactions, wherein the drug is expected to present inside and on the surface of the particles, the total drug content in the nanoparticles is estimated instead of encapsulation efficiency. The in vitro drug release profile of DTG-loaded Chitosan NPs in 0.1 N HCl medium showed comparatively faster and steady drug release than the pure drug (Supplementary Fig. 3). The in vitro dissolution study of the nanoparticles in buffer pH 1.2 was mainly focused since the chitosan nanoparticles formulation was developed for milk/food admixture for pediatric patients and is recommended for administration through the oral route. The release pattern of the drug in the stomach environment is mimicked in this study and also the influence of chitosan polymer as it is freely soluble acidic environment is evaluated. Enhanced solubility of chitosan-based nanoparticles in acidic pH of the stomach would facilitate the dissolution and bioavailability, which ultimately improve the drug absorption to reach the systemic circulation^20^. The drug release experiments of the nanoparticles were carried out in other media also (distilled water, phosphate buffer pH-6.8, and 0.1 N HCl media in the presence of digestive enzymes like pepsin and pancreatin, and the comparative results were published earlier^19^. The time taken to release 30%, 50%, and 80% (T_30_, T_50,_ and T_80_, respectively) of the drug from the NPs in the 0.1 N HCl medium was calculated based on the drug release profile. The time taken to release 80% of the drug was faster (6 h) for DTG-loaded Chitosan NPs than the pure drug (20 h). The result showed significant enhancement in the dissolution of DTG after encapsulating inside the Chitosan NPs. The release data were fitted into various kinetic models, which depicted Korsemeyer Peppas (KP) model as the best-fit drug release kinetics (Supplementary Fig. 3). Therefore, the mechanism of drug release from the nanoparticles could be correlated to the processes considered in the KP model such as diffusion of water into the particles, swelling of the particles due to aqueous entry, formation of gel, diffusion of the drug out of the membranes and dissolution of the drug into the media^21^.

### Cellular uptake study of NPs by CLSM

**Figure 3:**
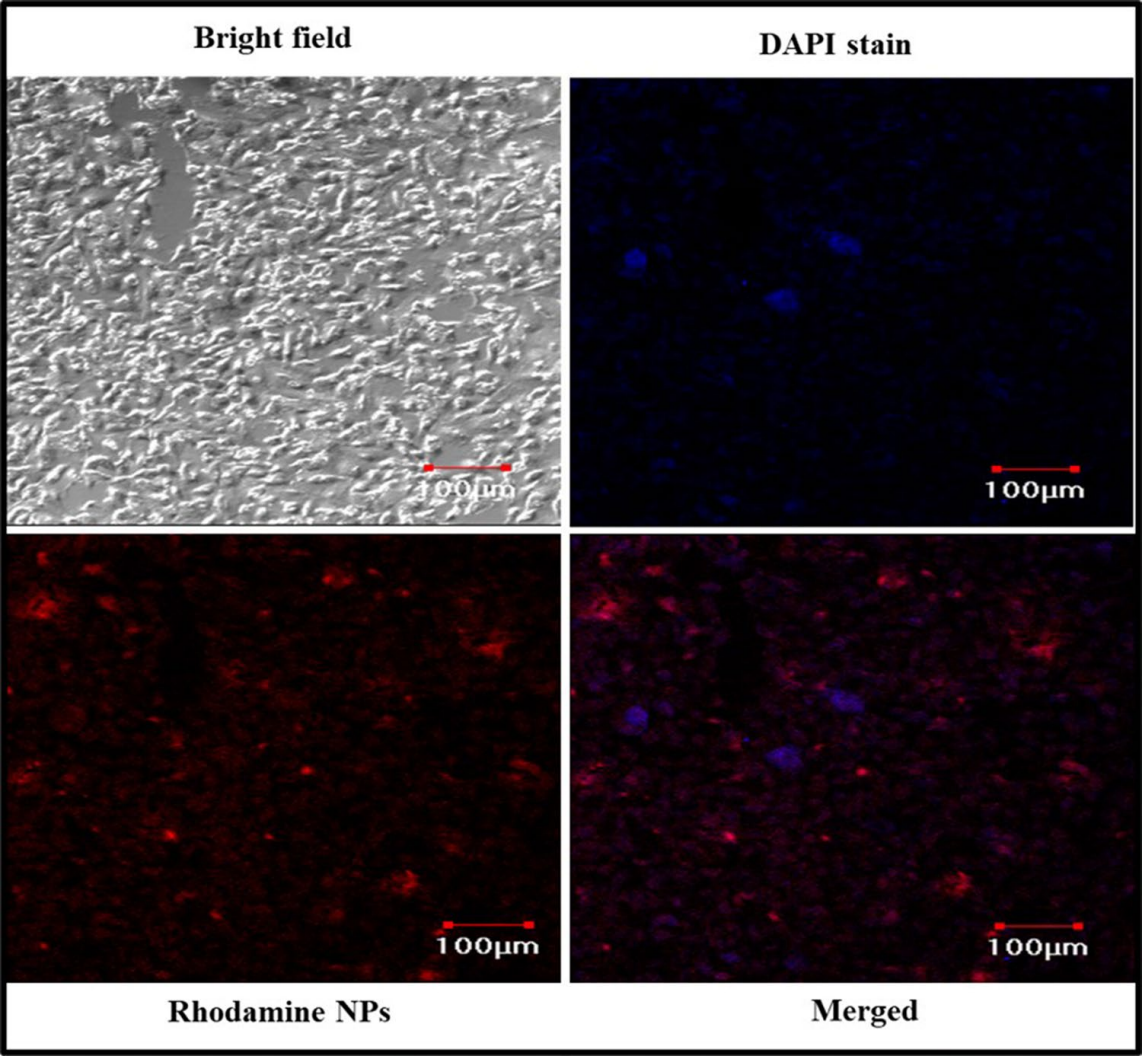
Cellular uptake study of nanoparticles in A549 cell lines imaged using confocal laser scanning microscope (CLSM).

The Rhodamine B nanoparticles showed the particle size distribution in the range of 154–750 nm with a polydispersity index of 0.740 mV. The nanoparticles possessed a positive surface charge (+ 4.15 mV) that was confirmed by zeta potential analysis (Supplementary Fig. 4). In the case of Rhodamine B nanoparticles, the identity peaks of the fluorescent dye were observed at 3688 cm^−1^, 2962 cm^−1^, 1550 cm^−1^, 1404 cm^−1^, 1246 cm^−1^, and 639 cm^−1^ for the molecular bonds O–H stretch, C–H stretch, C=C stretch, C–H bend, C–N stretch, and C = C bend, respectively^22^. The results showed that the dye remained chemically intact after the spray drying process to exhibit its fluorescent property (Supplementary Fig. 5). Recently, Salama et al., had demonstrated a similar experiment for the utilization of spray-dried Rhodamine B-loaded chitosan nanoparticles for the brain uptake study and also confirmed the fluorescence and chemical nature of the dye were not affected after the spray drying process^23^. The confocal imaging of the Chitosan NPs (loaded with Rhodamine marker) in lung epithelial cell lines (A549) showed that the nanoparticles have entered the cell and reached the nucleus (Fig. 3). The NPs were expected to utilize Clathrin-mediated Endocytosis (CME), where the amino group present in the Chitosan was responsible for the cellular entry^24^. The CLSM analysis confirmed that the nanoparticles had entered inside the nucleus, which was observed as a pink stain (DAPI nucleus staining dye) in the overlapped image^25^. The negatively charged drug was expected to masked with the positive charge on its surface due to the presence of Chitosan, which will aid in the enhanced cellular uptake. Since DTG could exhibit its mechanism of action inside the nucleus (inhibiting the strand transfer step), enhanced cellular uptake could be expedient for the drug to exhibit its maximum therapeutic activity^26^.

### In vivo pharmacokinetic and biodistribution studies of Chitosan NPs in mice model

#### Plasma biodistribution

**Figure 4:**
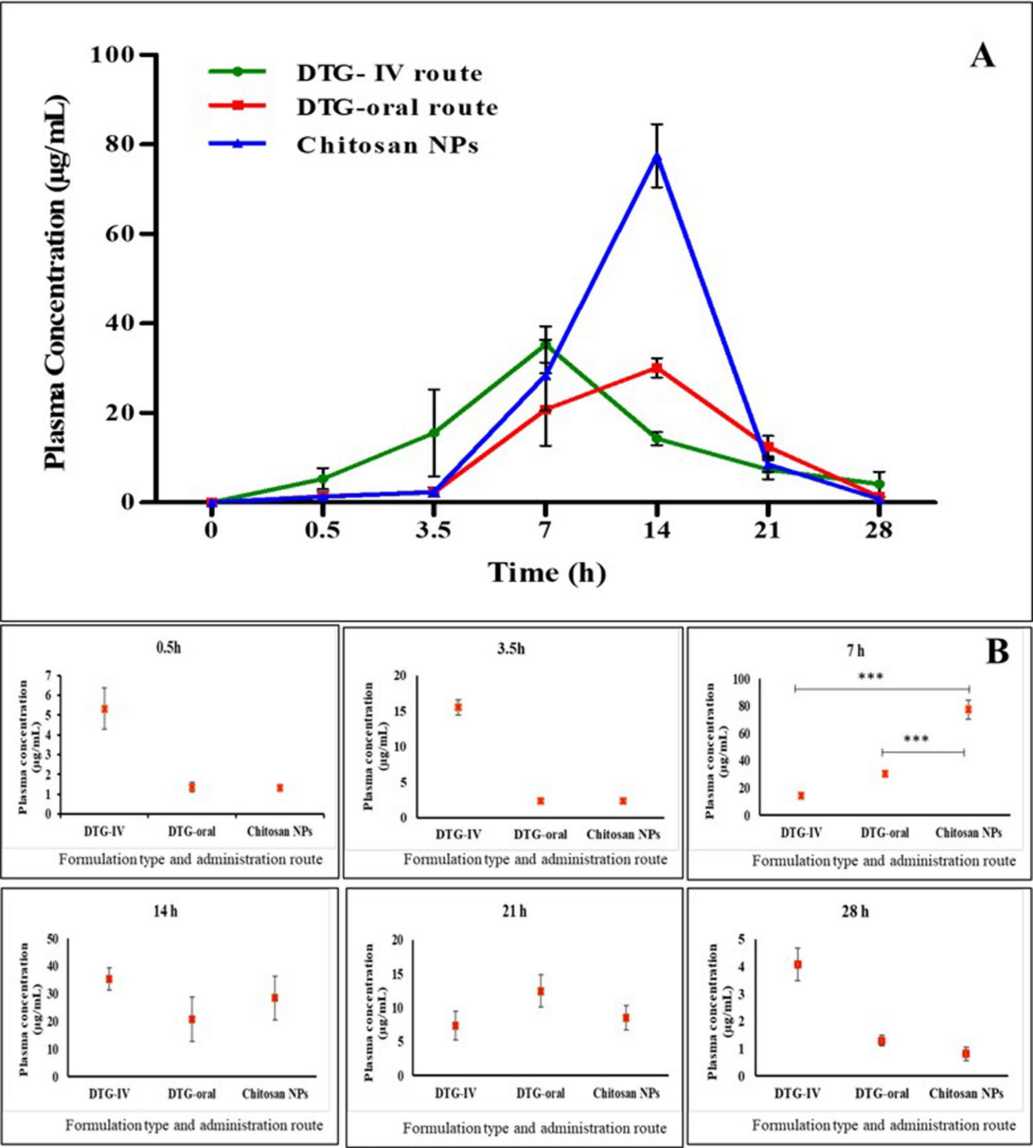
Comparative invivo pharmacokinetics of Dolutegravir and chitosan nanoparticles administered as milk admixture. (**A**) Plasma biodistribution of DTG after single oral administration. (**B**) Timewise plasma drug concentration of the Chitosan NPs compared with pure drug.

The in vivo pharmacokinetic evaluation of DTG-loaded Chitosan NPs through oral administration along with milk was carried out in Balb-C mice and compared against the pure drug. The plasma drug concentration–time profiles of pure DTG (by oral and IV route) and DTG-loaded Chitosan NPs were illustrated in Fig. 4, while the estimated pharmacokinetic parameters were summarized in Table 1. The blood and organ samples collected from different study groups at different time points (0.5, 3, 7, 14, 21, 28 h) were analysed using the developed HPLC method. The DTG-loaded Chitosan NPs and the pure drug given through the oral route showed the maximum plasma concentration of the drug (C_max_) at 7 h, followed by a gradual decrease in the plasma drug concentration (Fig. 4). The unprocessed DTG and the nano-formulation of DTG showed the C_max_ value of 29.33 ± 4.01 and 77.54 ± 7.93 µg/mL respectively (Table 2). At the 7th hour, there was a statistically significant escalation in the plasma drug concentration (p < 0.001) for the NPs compared to the pure drug. The DTG-loaded Chitosan NPs showed about a 1.6-fold increase in plasma drug concentration than the pure drug given through the oral and IV route. The DTG-loaded Chitosan NPs showed statistically (p < 0.01) higher AUC (678.3 ± 191.23 µg/mL) than the pure drug (405.29 ± 7 µg/mL). The absolute and the relative bioavailability of DTG-loaded Chitosan nanoformulations were calculated to be 165 ± 8.14% and 167.30 ± 13.63%, respectively. When Lee et al.^27^ had quantified the plasma biodistribution of DTG in mice models after single oral administration in the form of suspension, the C_max_ (50 µg/mL) was reached at 3.5 h, followed by a gradual decrease in the drug concentration in the plasma. In our study, the C_max_ was achieved at 7 h, due to the sustained release of the drug from matrix chitosan nanoparticles and the presence of milk as a vehicle. The effect of milk could be well correlated to our in vitro drug release studies conducted in presence of milk and enzymes. During the in vitro drug release studies, to estimate the effect of milk on drug release time, the drug/nanoparticles were mixed with milk, and the release study was carried out in a 0.1 N HCl medium. There was no significant difference observed in the drug release profile with and without milk. However, in the presence of enzymes (pepsin and pancreatin), there was a slight decrease in the release rate of the drug. The multivalent cations of calcium and magnesium present in milk were expected to chelate with the drug in presence of GI luminal fluids, which resulted in the formation of complexes thereby consuming more digestion time^28^. Hence, it could be concluded that the presence of milk and the in vivo milieu (enzymes and digestive fluids) caused the delay in drug absorption, leading to a high T_max_ of 7 h (time to reach the C_max_) in our experiment. Song et al. had analysed the pharmacokinetics of DTG in healthy subjects for 24 h when administered along with a Calcium supplement. The results suggested that DTG could be co-administered along with Ca^2+^ if consumed with a meal, or it should be separated if given under fasting conditions for the best efficacy and absorption of DTG^29^. The concentration of the drug in the plasma was notable till 21 h post administration of the single dose. Based on the in vitro HIV inhibition assay in C8166 cell lines infected with HIV_IIIB_ strain, the EC_50_ value of Dolutegravir-loaded Chitosan NPs was predicted to be 0.82 ± 0.71 ng/mL. Based on the in vivo study, the concentration of the drug in the plasma at 21 h for the Chitosan NPs was 8.53 ± 1.2 µg/mL, which is far higher than the EC_50_ value. Hence, the developed nanoformulation could be considered suitable for once-daily administration for paediatrics.

**Table 1:** Pharmacokinetic parameters of the treated animals with pure DTG and Chitosan NPS.

S. no.	PK parameters	DTG (IV route)	DTG (oral route)	Chitosan NPs
1	t_1/2_ (h)	10.54 ± 1.1	11.72 ± 1.6	13.55 ± 0.69
2	C_max_ (µg/mL)	29.33 ± 4.01	30.15 ± 8.06	77.54 ± 7.93
3	T_max_ (h)	7	7	7
4	K_e_ (h^−1^)	0.065	0.059	0.052
5	K_a_ (h^−1^)	0.26	0.48	0.53
6	Clearance (L/h)	0.49 ± 0.03	0.48 ± 0.33	0.29 ± 0.08
7	V_d_ (L/kg)	1.87 ± 0.81	4.36 ± 0.78	7.67 ± 1.26
8	MRT	12.07 ± 0.05	10.07 ± 0.93	14.74 ± 1.2
9	AUC_0–24 h_ (µg/mL)	411.02 ± 91	405.29 ± 7	678.3 ± 191.23
10	AUMC_0–24 h_ (µg/mL)	4357.9 ± 990	5704.6 ± 892.3	10,003.1 ± 1269
11	Absolute bioavailability (%)	–	–	165 ± 8.14
12	Relative bioavailability (%)	–	–	167.37 ± 13.63

**Table 2:** Hematology results of the treated blood samples.

Parameter	Control	DTG-IV	DTG-oral	Chitosan NPs
WBC (10^3^/µL)	14.20 ± 3.45	18.14 ± 10.24	17.81 ± 3.38	15.96 ± 7.26
Neutrophil (10^3^/µL)	4.22 ± 0.64	5.15 ± 1.74	5.32 ± 1.71	5.04 ± 2.25
Lymphocyte (10^3^/µL)	9.85 ± 2.79	12.83 ± 8.46	12.30 ± 1.67	10.77 ± 4.79
Monocytes (10^3^/µL)	0.10 ± 0.07	0.10 ± 0.07	0.15 ± 0.06	0.12 ± 0.09
Eosinophil (10^3^/µL)	0.04 ± 0.03	0.06 ± 0.08	0.04 ± 0.03	0.03 ± 0.03
Basophils (10^3^/µL)	0.00	0.01 ± 0.02	0.00	0.00
RBC (10^6^/µL)	10.17 ± 11.8	8.60 ± 2.47	9.69 ± 0.48	9.75 ± 1.05
HGB (g/dL)	14.47 ± 0.97	13.70 ± 2.95	15.10 ± 0.72	14.47 ± 1.27
HCT (%)	64.30 ± 5.02	55.83 ± 17.67	63.77 ± 1.96	58.80 ± 6.99
MCV (fL)	67.20 ± 3.55	64.47 ± 2.75	65.90 ± 4.10	60.27 ± 1.55
MCH (pg)	15.13 ± 10.7	16.23 ± 1.46	15.57 ± 0.15	14.87 ± 0.59
MCHC (g/dL)	22.50 ± 0.82	25.27 ± 3.33	23.67 ± 0.15	24.67 ± 0.95
RDW-CV (%)	17.03 ± 11.4	15.37 ± 1.11	15.47 ± 1.58	16.73 ± 1.99
RDW-SD (fL)	46.80 ± 21.6	40.60 ± 4.25	41.73 ± .90	41.17 ± 4.45
PLT (10^3^/µL)	551.00 ± 31.6	617.00 ± 27.89	674.67 ± 42.91	656.00 ± 19.52
MPV (fL)	5.90 ± 0.56	6.03 ± 0.15	5.97 ± .06	6.13 ± 0.21
PDW	15.07 ± 0.15	15.23 ± 0.32	15.17 ± 0.15	15.03 ± 0.06
PCT (%)	0.33 ± 0.20	0.50 ± 0.18	0.52 ± 0.07	0.40 ± 0.12

#### Organ biodistribution

**Figure 5:**
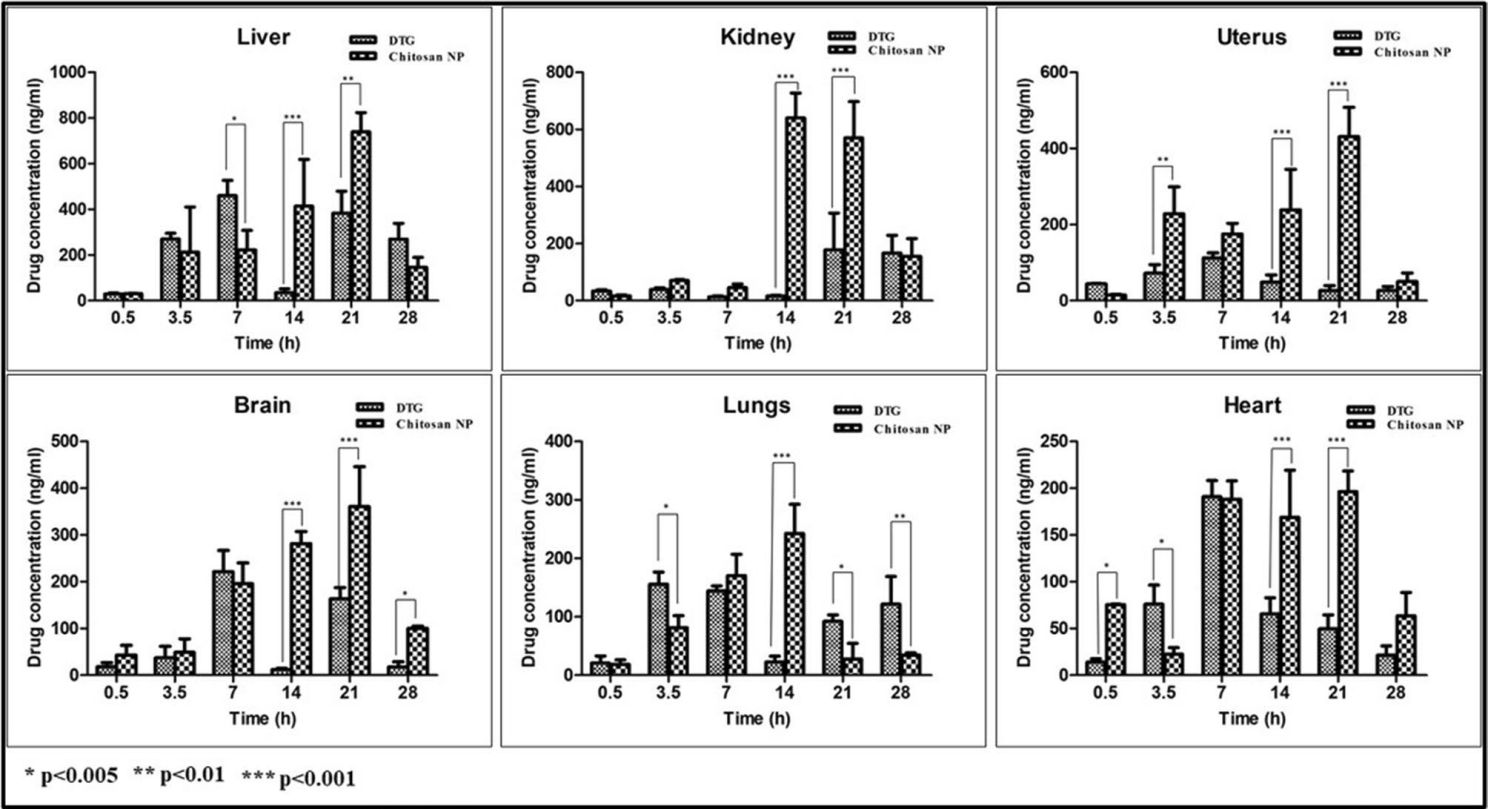
Organ biodistribution of the drug in Chitosan NPS compared with pure drug in the treated animals.

The DTG-loaded Chitosan NPs displayed higher AUC in all the organs when compared to the pure DTG given through oral and IV routes (Supplementary Table 4). For the study groups treated with pure DTG by oral and IV routes, the C_max_ reached in 7 h in all the organs like lungs, liver, brain, heart, and uterus (except kidney, where C_max_ reached after the first half-life), followed by a gradual decrease in the concentration further (Fig. 5). The group of animals administered with DTG-loaded Chitosan NPs displayed a steady-state increase in the concentration of the drug in all the organs, wherein the C_max_ was reached at 21 h. Since HIV is a sexually transmitted disease, the concentration of the drug in genital organs like the uterus could be considered significant^30^. The NPs exhibited higher AUC in the uterus than the pure drug. Because of the fairly higher levels of DTG in blood after the oral administration of NPs, the increased accumulation of DTG in the brain proposes a non-saturable uptake of NPs from the blood to the brain. These observations are supported by previous studies suggesting that Chitosan NPs may be able to cross the blood–brain barrier^31^. In the kidney, liver, heart, and lungs, there was a significant amount of DTG present for the time span of 28 h after oral administration. The bioaccumulation profile of the drug showed the following order, Liver > Kidney > Uterus > Brain > Heart > Lungs. The clearance of the NPs was found to be lower than the unprocessed DTG (Supplementary Table 2) given through the oral route. Also, the volume of distribution (V_d_) was higher for the DTG-loaded Chitosan NPs (7.67 ± 1.26 L) when compared with the orally-administered pure DTG (4.36 ± 0.78 L). It could be concluded that the presence of chitosan intended the slow release of the drug in blood and hence the time taken to reach (T_max_) the C_max_ in the organ is higher compared to the pure drug, and the higher V_d_ of the NPs caused the fairly higher distribution of DTG in the organs than the pure drug.

#### Histomorphology of organ samples treated with Chitosan NPs

**Figure 6:**
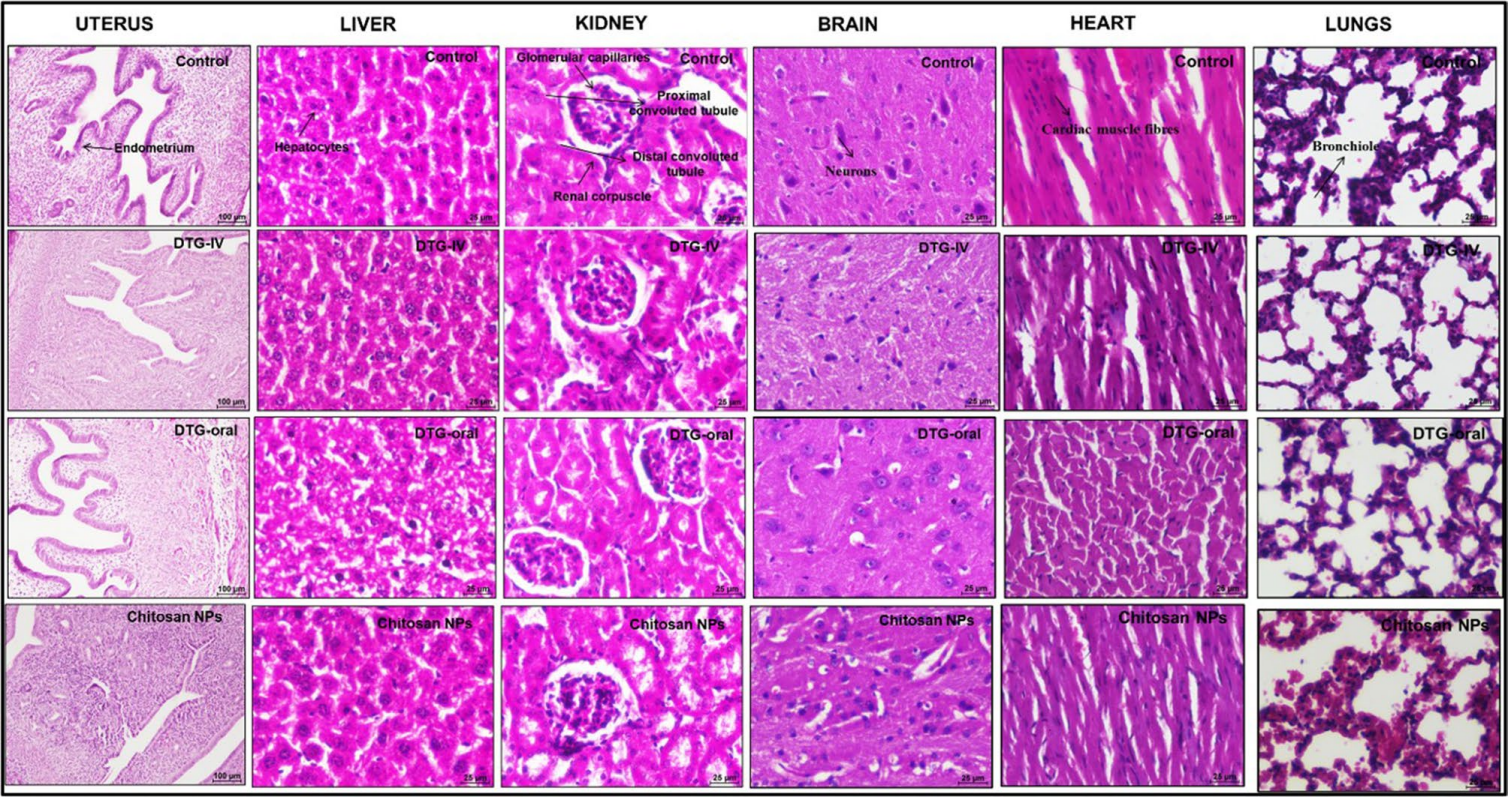
Histomorphological analysis of the organs of treated animals with Chitosan NPs nanoformulation compared with the control (untreated).

The histomorphological analysis of the organs like liver, kidney, heart, brain, liver, and uterus was performed after the administration of DTG-loaded Chitosan NPs along with milk, and the results were presented in Fig. 6. The results of the NPs treated groups of animals were compared with the animals administered with pure DTG (by oral and IV administration) and also with control (untreated) samples. Although there was a considerable distribution of the drug/NPs in all these organs, there was no significant difference in the morphology and pathology of the tissues, which confirmed that the administration of the DTG as nanoparticles could be considered non-toxic to the organs.

#### Haematology of blood samples treated with Chitosan NPs

Further, the hematological studies were performed to evaluate the deviations in the levels of red blood cells, white blood cells, hemoglobin, haematocrit, mean corpuscular volume (MCV), mean corpuscular haemoglobin concentration (MCH), red blood cell distribution width (RDW), neutrophils, lymphocytes, monocytes, eosinophils, basophils, and platelet count (PCV), platelet distribution width (PDW). All the parameters presented no statistically significant difference between the control (untreated) and the experimental mice groups (Table 2), which confirmed that both the oral and IV administration of DTG and the DTG loaded Chitosan NPs are non-toxic to the blood cells.

## Methods

### Physical characterization of Chitosan NPs

Particle size analysis was accomplished using the dynamic light-scattering principle. About 10 mg of the DTG-loaded Chitosan NPs were mixed with 1 mL of distilled water and sonicated for about 10 min for complete dispersion. The dispersion was placed in a two-way cuvette and the sample was analysed using a zeta-sizer (Malvern nano series ZS, UK)^32^. Further, the synthesized NPs were analysed through Scanning Electron Microscope with a focussed beam of electrons to compare the texture of the formed particles. A small amount of sample was taken for the microscopic analysis with magnification ranging from × 3000 to × 1,00,000. The sample positioned on the stub was sputter-coated through a thin film of gold using an auto sputter fine coater (JFC 1600, JEOL, Japan) prior to imaging. The sputter-coated sample was introduced into the sample chamber and imaging was performed at an accelerating electrical energy of 3 kV^33^.

### Chemical characterization of Chitosan NPs

The prepared NPs were subjected to FTIR analysis to study the chemical stability of the drug in presence of chitosan polymer after the spray drying process. The analysis was performed through the KBr pellet technique, wherein about 5 mg of drug-loaded Chitosan NPs were blended with the formerly dried saturated KBr. Afterward, the pellets were shaped using a hydraulic pressure unit at 53.62 kp N/cm^2^. The obtained pellets were shifted to the scanning stub and scanned at the wavenumber ranging from 4000 to 400 cm^−1^^34^. The thermal behavior of the pure DTG and the drug-loaded Chitosan nanoparticles were measured by employing the principle of differential scanning calorimetry (Q100, TA instrument). Approximately, 16–20 mg of the DTG-loaded Chitosan NPs and the pure drug were individually placed in aluminium pan, which was positioned on the sample holder and heat flow was applied at 80 °C/min up to 900 °C, keeping palladium compound as standard in a nitrogen atmosphere^35^.

### In-vitro drug release and kinetics of Chitosan NPs in 0.1 N HCl media

The in vitro drug release study of the DTG-loaded Chitosan NPs was compared with the pure DTG in 0.1 N HCl pH 1.2 by dialysis bag method (Dialysis membrane Himedia, cut-off value—12–14 kDa). About 10 mg of pure drug and a similar dose equivalent amount of nanoparticles were taken for the drug release experiments. The test samples were mixed with 0.5 mL of the corresponding media, placed inside the dialysis bag, and immersed in 10 mL media in a vial. The temperature and magnetic stirring speed of the sample were retained at 37 °C ± 2 °C and 200 rpm, respectively throughout the 24 h study period. At every predetermined time point, the 10 mL samples were collected and immediately replaced with the fresh corresponding medium. The concentration of the drug in each sample was quantified by a UV–Visible spectrophotometer. The UV visible spectrophotometer (UV 1800, Shimadzu, Japan) was initialized with λmax of 260 nm. The absorbance of the test solution was recorded using the quartz cuvette (volume-3.5 mL, path length-1.5 cm) and the calculations were made based on the standard calibration curve by following the Beer Lamberts law. The obtained release data were fitted into the various drug release kinetics namely zero order, first order, Higuchi, Hixson–Crowell, Korsmeyer–Peppas, Makoid Banakar, Gompertz, Hopfenberg, and Baker Lonsdale. The R^2^ value and the n-value obtained from these models were compared to conclude the perfect fitting model for each trial. The drug release modelling was analysed using DD solver Software^36^.

### Cellular uptake study of NPs by CLSM

The A549 cell lines were utilized for the cellular uptake study as they are derived from the human respiratory epithelium and are the most popular category of Type II alveolar epithelial cells (AECII), which play a vital role in the antigen presentation to T-lymphocytes (CD4 cells)^37^. Since HIV primarily infects the CD4 cells that help in coordinating the immune response with the help of other immune cells like macrophages, B-lymphocytes, etc., the A549 cell lines were selected for the cellular uptake study of the anti-HIV nanoformulation. Previously, Kim et al., reported that the A549 cell lines had the ability for rapid uptake of the nanoparticles due to their high adherence property^38^. A549 cells were grown at 37 °C and 5% carbon dioxide in Dulbecco’s modified Eagle medium (DMEM) supplemented with 10% fetal bovine serum in cell culture dishes. The cells were routinely passaged at 90–95% confluency. Firstly, the DMEM used to incubate A549 cells was removed and the cells were washed once with PBS. During the in vitro optimization studies, the Chitosan NPs with the drug and polymer ratio of 1:1 had shown better response in terms of particle size, drug content and drug release rate, etc.^19^. Hence the Rhodamine B dye and the Chitosan polymer were taken in a 1:1 ratio and dissolved in 1% acetic acid and the solution was spray-dried at 140 °C. Further, the cells were treated with DMEM solutions dispersed with Rhodamine-B loaded Chitosan nanoparticles (1 mg/mL), that were washed (3 cycles) before the study to remove the surface-associated and the free dye in the nanoformulation and incubated for 1 h at 37 °C in an atmosphere of 5% carbon dioxide. The nucleus staining dye DAPI was added to the coverslips before washing the cells with PBS. After treatment with the samples for 1 h, the cells were imaged using a confocal laser scanning microscope (Olympus FV-1000, Japan) using a Rhodamine B filter (Excitation wavelength 553 nm and Emission wavelength 627 nm) and the images were captured at × 10 magnification^39^.

### HPLC method development for Dolutegravir

The pharmaceutical grade working standards of Dolutegravir were obtained as a gift from MSN Laboratories Hyderabad, India. The standard calibration of the pure drug in the mobile phase (Water: Acetonitrile: Methanol in 20:40:40 ratio with 0.2% formic acid) and mice plasma was plotted using the developed method. The plasma samples of untreated normal Balb/c mice were obtained from the Central Animal Facility, SASTRA Deemed University (Approval ID: 585/IAEC/RPP), and the plasma samples were spiked with known concentrations (1, 2, 3, 4, 5 and 10 µg/mL) of DTG and allowed to incubate at room temperature for 15 min. The detection was carried out at 260 nm with an injection volume of 20 μL, wherein the flow rate of the mobile phase was maintained at 1.0 mL/min at the column temperature of 35 °C. Moreover, the validation of the analytical method was also performed in accordance with the procedure referred to in the International Council on Harmonization (ICH) guidelines^40^.

### Quantification of Dolutegravir in mice blood and organ samples through HPLC analysis

After validating the developed HPLC method, the same was utilized to quantify the drug present in the plasma and organ (lungs, liver, kidney, heart, brain, and uterus) samples collected at predetermined time points. A total of 114 animals (25–30 g) were acquired from the Central Animal Facility (CAF), SASTRA University, Thanjavur after obtaining the ethical approval from Institutional Animal Ethics Committee (IAEC). The animals were randomly divided into four groups (n = 6) for this study. All animals were housed in individual cages in a temperature-controlled facility. The surgical procedures were approved by IAEC at SASTRA University (585/SASTRA/IAEC/RPP). The experiments were performed following the guidelines issued by the Committee for the Purpose of Control and Supervision of Experiments on animals (CPCSEA), Ministry of Environment and Forest, Government of India and Animal Research: Reporting of In Vivo Experiments (ARRIVE). The 2 groups of mice were orally treated with pure DTG and DTG-loaded Chitosan NPs at the dose equivalent to 10 mg/kg^41^ along with milk. The 3rd group of animals was treated with DTG at the same dose through an intravenous route to elucidate the absolute bioavailability and the 4th group of animals was reserved as untreated control for comparison. The blood and organ samples were withdrawn from all the experimental mice at 0.5 h, 3.5 h, 7 h, 14 h, 21 h, and 28 h after euthanizing the animal using CO_2_ inhalation. The concentration of the drug in each sample was determined by the developed HPLC method, followed by the pharmacokinetic parameters and biodistribution pattern evaluation^42^.

### Histomorphological analysis of organ samples

The organs of the test animals were excised and fixed in a 10% formalin solution. Before embedding in paraffin wax, the tissue samples were dehydrated in an Automatic Tissue Processor (Leica TP1020, Leica Microsystems, Germany) by transferring through a series of gradually increasing percentages of alcohol. The tissue samples were embedded in paraffin using an embedding machine (EG1150 H&C, Leica Microsystems, Germany), sectioned using a microtome (Rotary Microtome Leica RL2125RT, Leica Microsystems, Germany), and stained with haematoxylin and eosin. These samples were viewed under a light microscope (Nikon, eclipse, C1-L, Japan) to determine the pathological changes or toxic reactions in the cells, and the tissue reaction was rated as per ISO 10993-6^43^.

### Haematological analysis of blood samples

The complete blood cell (CBC) count was performed for the blood samples of the treated animals after 28 h of administration of the test substances. The counting was made following the method of Beckman Coulter. It measures the changes in electrical resistance produced by non-conductive particles suspended in an electrolyte. The blood sample from the test animals was collected in EDTA tubes and was further suspended through a small orifice along with an electric current. At the sensing end, the suspended particle displaces its volume of electrolyte which was measured as the voltage pulse by the Beckman Coulter. The height of the voltage pulse in proportion to the volume of the blood cell. The white blood cells were quantified based on the VCS technology, where the individual cell volume (V), conductivity (C), and laser light scatter (S) were measured and the scattergram plotted the cell based on the above measurements^44^.

### Statistical analysis

All the results were presented in the form of mean ± SD, and statistically significant differences between treatments were calculated using the student’s t-test and ANOVA test using GraphPad Prism version 5 software.

## Conclusion

The present investigations deal with the synthesis and characterization of DTG-loaded Chitosan nanoparticles through spray drying technology. The feasibility of administration of DTG as nanoformulation along with milk has been experimented with and validated through the in vivo study using Balb-C mice models. The drug was quantified by the developed HPLC method using acetonitrile, methanol, and water as mobile phases. The plasma and the organ biodistribution of the drug were significantly higher when administered as nanoformulation than the pure drug. The coadministration of the drug along with milk has not affected the absorption coefficient in vivo, however, the time taken to reach the maximum concentration [Tmax] was delayed. The distribution of the drug in the uterus and brain was significantly higher when administered as nanoformulation. The histomorphological and haematological analyses of the organ and blood samples respectively showed no toxicity associated with the administration of the nanoparticle. Hence, it could be concluded that DTG-loaded Chitosan nanoparticles administration would be beneficial for the paediatric anti-HIV therapy, especially as milk admixture whereby the treatment adherence is augmented.

## Supplementary Information

Supplementary Information.

## Data Availability

The datasets generated and/or analyzed during the current study are not publicly available due to (Funded project and No permission from the same) but are available from the corresponding author at Pharmaceutical Technology Laboratory, ASK-II, Lab No: 214, School of Chemical & Biotechnology, SASTRA Deemed-to-be-University, Thanjavur-613401, Tamil Nadu, India or through email—vedhahari@scbt.sastra.edu.
