# Supplementary material for: In-vivo pharmacokinetic studies of Dolutegravir loaded spray dried Chitosan nanoparticles as milk admixture for paediatrics infected with HIV
Source: Sci Rep. 2022 Aug 16;12:13907. doi: 10.1038/s41598-022-18009-x (PMC9381509; doi:10.1038/s41598-022-18009-x)
Supplement: Supplementary file 1 — Supplementary Information. [file 41598_2022_18009_MOESM1_ESM.docx]

***In-vivo* Pharmacokinetic Studies of Dolutegravir loaded Spray Dried Chitosan Nanoparticles as Milk Admixture for Paediatrics Infected with HIV**

Priya Dharshini K^1^, Ramya Devi D^1^, Banudevi S^2^, Vedha Hari B Narayanan^1,*^

1. Pharmaceutical Technology Laboratory, ASK-II, Lab No: 214, School of Chemical & Biotechnology, SASTRA Deemed-to-be-University, Thanjavur-613401, Tamil Nadu, India.

2. Centre for Nanotechnology and Biomaterials, School of Chemical & Biotechnology, SASTRA Deemed-to-be-University, Thanjavur-613401, Tamil Nadu, India.

[*vedhahari@scbt.sastra.edu](mailto:*vedhahari@scbt.sastra.edu)

Supplementary Figure 1: Comparative HPLC Chromatograms of Dolutegravir developed using a mixture of water, acetonitrile and methanol in the ratio of 20:40:40 with 0.2% formic acid as the mobile phase [A-E] Dolutegravir (100 ng-500 ng/ml); [F-I] Plasma samples spiked with DTG (1 µg/ml – 5 µg/ml); [J] Calibration curve for DTG in mobile phase; [K] Calibration curve for plasma spiked with DTG


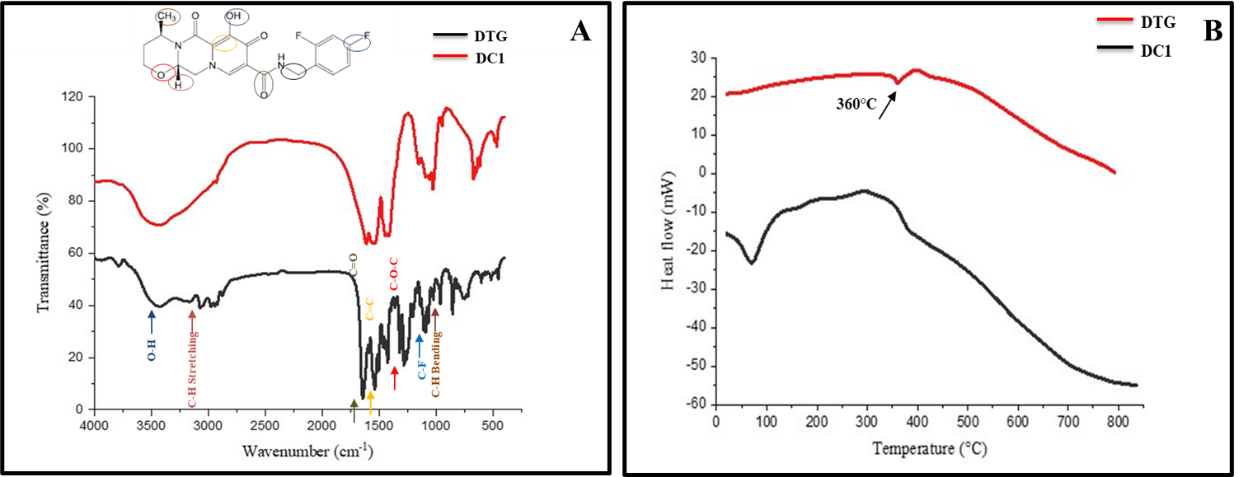


Supplementary Figure 2: Thermal and chemical characterization chitosan nanoparticle loaded with Dolutegravir [A] Fourier transform infrared spectroscopy to determine the chemical stability of DTG in Chitosan NPs post spray drying process; [B] Effect of spray drying process on the thermal behaviour of DTG in Chitosan NPs

**
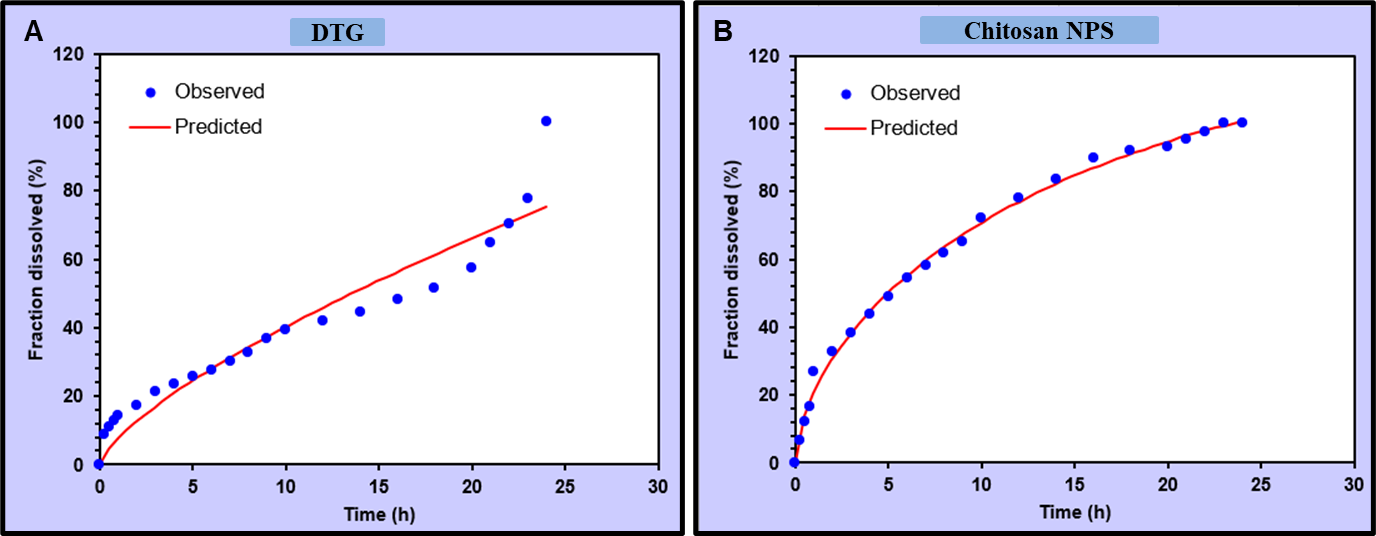
**

Supplementary Figure 3: *Invitro* drug release profile of Chitosan NPs in 0.1 N HCl media fitted into Korsemeyer Peppas model of release kinetics: [A] DTG: [B] Chitosan NPs


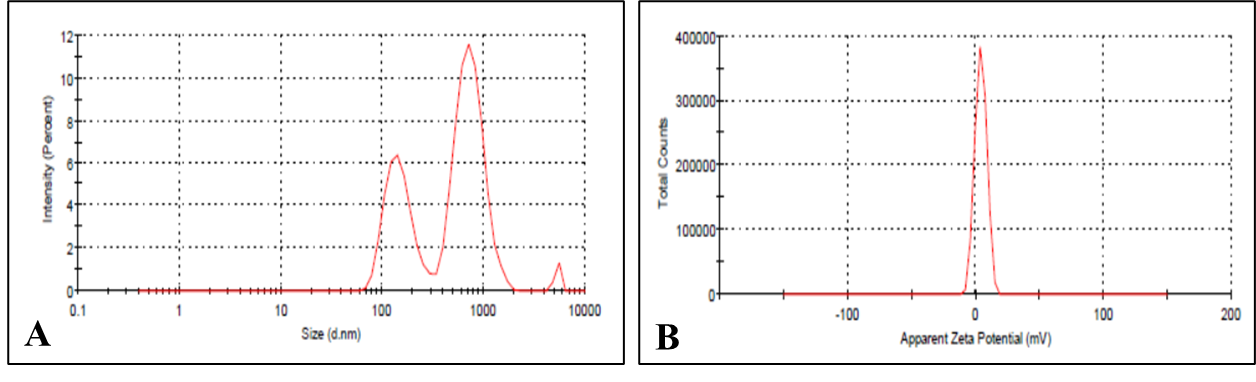
Supplementary figure 4: A- Dynamic light scattering spectroscopy for determining particle size distribution; B- Zeta potential analysis by charge conductivity principle.


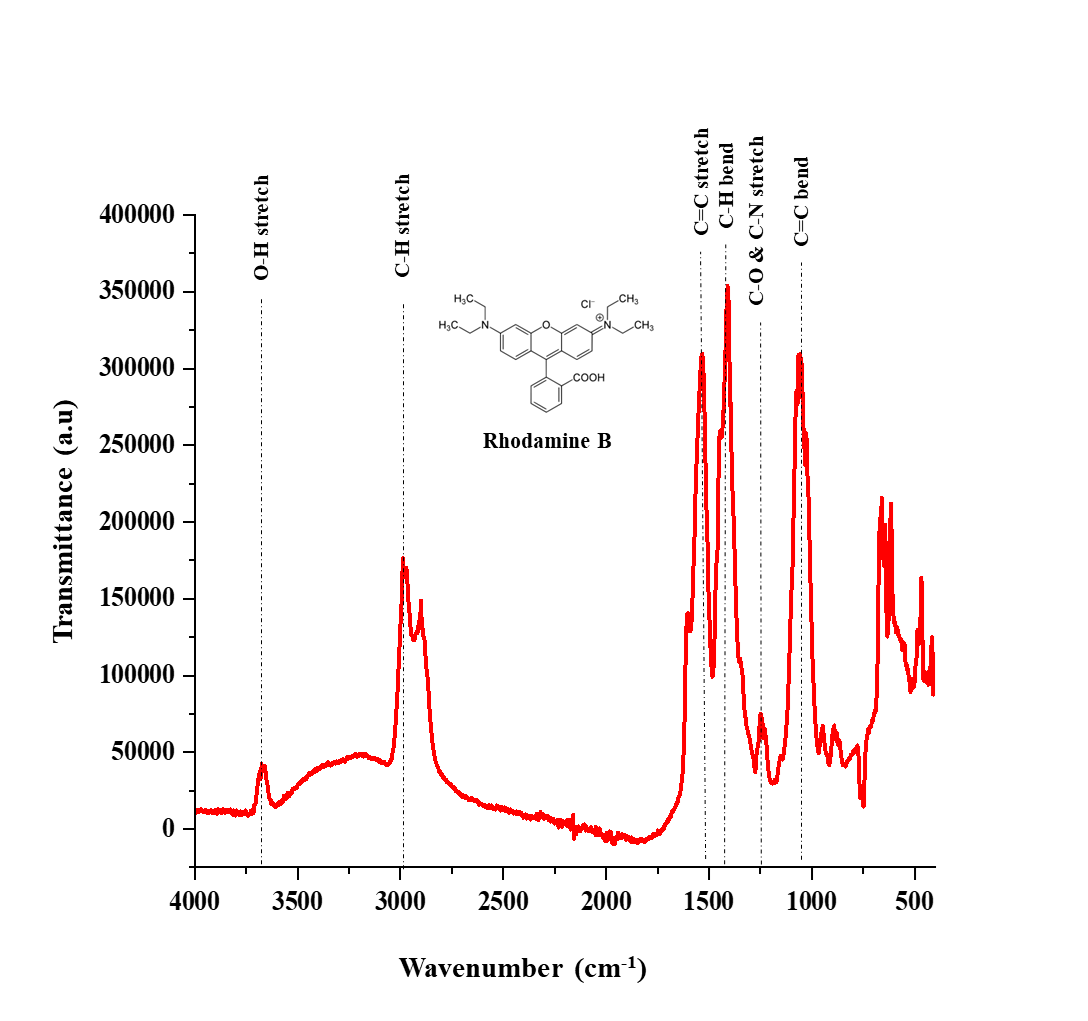


Supplementary figure 5: FTIR analysis to confirm the chemical stability of Rhodamine-B after the spray drying process.

| System suitability | | |
| --- | --- | --- |
| **Parameter** | **Results** | **Limits** |
| RSD of peak area | 1.047 | <2.0 for n ≥ 6 |
| RSD of retention time | 0.47 | <1.0 for n ≥ 6 |
| Column Efficiency | 8.22 | - |
| USP plate count (N) / m | 3289 | >2000 |
| USP peak asymmetry factor (A_s_) | 1.43 | A_s_ >1- tailing; A_s_ <1 Fronting |
| USP tailing factor (T) | 1.407 | T < 2 |
| USP resolution (R | 2.79 | R > 2 |
| **Accuracy** | | |
| **Parameter** | **Amount added** | **% of recovery** |
| Low level | 4 | 99.56 ± 0.3 |
| Mid-level | 40 | 99.43 ± 0.5 |
| High level | 400 | 99.24 ± 0.3 |
| **Precision** | | |
| **Repeatability** | | |
| RSD of retention time | | 0.31 |
| RSD of peak area | | 1.26 |
| **Reproducibility** | | |
| RSD of retention time | | 0.39 |
| RSD of peak area | | 1.35 |
| **Intermediate precision** | | |
| RSD of retention time | | 0.42 |
| RSD of peak area | | 1.59 |
| **Linearity and range** | | |
| **Parameter** | | **Results** |
| Linearity range | | 100 ng/ml – 400 µg/ml |
| Correlation coefficient | | 0.9995 |
| Slope | | 95.365 ± 3.2 |
| Y intercept | | 1269.1 ± 295 |
| LOQ | | 24.91 ng/ml |
| LOD | | 8.25 ng/ml |

**Supplementary Table 1:** Method validation parameters of the developed HPLC method for the quantification of DTG in mice plasma

| Formulation | DTG-IV | DTG-oral | Chitosan NPs |
| --- | --- | --- | --- |
| Organ | (µg/mL) | | |
| Uterus | 5.5 ± 1.2 | 1.5 ± 0.4 | 6.5 ± 3.1 |
| Lungs | 4.3 ± 0.68 | 2.5 ± 0.4 | 3.2 ± 1 |
| Liver | 4.6 ± 1.2 | 7.2 ± 1.4 | 10.49 ± 2.8 |
| Kidney | 3.8 ± 1.3 | 2.2 ± 0.4 | 9.5 ± 3.6 |
| Heart | 2.0 ± 0.38 | 2.1 ± 0.4 | 3.9 ± 0.7 |
| Brain | 2.4 ± 0.33 | 2.6 ± 0.5 | 6.1 ± 1.1 |

**Supplementary Table 2:** AUC of biodistribution of DTG in organs
